# Liposuction

**Published:** 2008-10

**Authors:** Lakshyajit D. Dhami

**Affiliations:** Laser, Aesthetic and Plastic Surgeon, Vasudhan Arjin Cosmetic and Laser Surgery, Mumbai, India

**Keywords:** Obese, liposuction, megaliposuction, super-wet technique

## Abstract

Advent of the tumescent technique in 1987 has allowed for safe contouring in ambulatory single session liposuction under regional or general anaesthesia. Safety and aesthetic issues define MegaLiposuction to be in Volume in litres of more than 10% of Body weight in Kgs. 870 cases of liposuction were performed between September 2000 and August 2008. In (65%) cases, the total volume of aspirate was greater then 5 liters. (Range: 5 to 25 liters). In 24% cases, the large volume liposuction was combined with a limited or a total block lipectomy. Regional anaesthesia with conscious sedation was preferred except where liposuction was for above the subcostal region (the Upper Trunk, Lateral Chest, Back, Gynaecomastia, Breast, Arms and Face) or when the patient so desired. Tumescent infiltration with Lactated ringer, adrenalin, triamcinalone and hyalase was made in all cases. This approach has clinically shown less tissue edema in the post operative period than when the conventional physiological saline was being used in place of Ringer Lactate. The amount injected varied from 1,000 ml to 12,500 ml depending on the size, site and area. Local anesthetic was included only to the terminal portion of the tumescent mixture while infiltrating the sub-costal regions, or when above costal region was combined with below costal region being anaesthetized with Spinal Anaesthesia. The aspirate was restricted to the unstained white / yellow fat and the amount of fat aspirated did not have any bearing to the amount of solution infiltrated. There was no major complication. Blood transfusion was given only on one occasion when the patient had been on aspirin and had also received Low Molecular weight Heparin intra-operative. The hospital stay ranged from 8 to 24 hours for liposuction as well as for liposuction with a lipectomy. Serous discharge from access sites, sero-sanguinous fluid accumulation requiring drainage were necessitated in less than 10% cases. Minor re-contouring touch ups were requested in 5% cases. Early ambulation was encouraged for mobilization of third space fluid shifts to expedite recovery and to prevent deep vein thrombosis. More than 10% patients were operated on for Liposuction of other areas, after a gap of 7 days to 6 months. Meticulous perioperative monitoring of systemic functions ensures safety in tumescent megaliposuction for the obese and rewarding results can be achieved in a single sitting.

## HISTORY

Advent of the tumescent technique in 1987[1] has allowed for safe contouring in ambulatory single session liposuction under regional or general anaesthesia. Safety and aesthetic issues define MegaLiposuction to be in Volume in litres of more than 10% of Body weight in Kgs.

In a quest for everlasting beauty, man has made attempts to defy the ageing process by making use of all the available material at hand. He has used various oils, chemicals, minerals and camouflage techniques The earliest of the surgical techniques at enhancing physical appearances involved amputation of the unsightly deformity.

The concept of removing excess fat from localized body sites to achieve similar gains is credited to Charles Dujarrier, who in France,[2–4] attempted to remove subcutaneous fat using a uterine curette on calves and knees of a ballerina in 1921. An inadvertent injury of the femoral artery led to amputation of the dancer's leg. This unfortunate complication arrested further progress in this field and but it was a valiant attempt at the time.[5]

Schrudde in 1964[4] revived interest in this procedure and extracted fat from the leg, gaining access through a small incision with a curette, but was faced with a daunting task of managing the difficult haematoma and seroma that resulted from this technique. Subsequently, Pitanguy[6] favoured an en bloc removal of both fat and skin to remove excess thigh adiposities, but the extensively noticeable incisions did not allow the technique to become popular.

## MODERN LIPOSUCTION

Modern liposuction began with the technique and instruments of Giorgio Fischer, and his father Arpad Fischer, both, Gynaecologists from Rome, Italy, in 1974.[4] They developed their instruments themselves and their early cannulae contained a cutting blade within them. They eventually developed a blunt hollow canula connected to a suction apparatus and published their results in 1976. They developed the technique of crisscross tunnel formation from multiple access sites with their improved cannulae and demonstrated good results with fewer complications. In 1978 Kesselring and Meyer[7] published results of a sharp curettage aided by suction. The technique did not gain much acceptance in view of the significant complications.

Pierre Fournier[8] of Paris France, improvised on the Fischer's' liposculpture technique and was the initial advocate of the ‘dry technique’ in which no fluids were infiltrated prior to liposuction. He went on to become an authority in liposuction and fat transplantation and later promoted the benefits of tumescent anaesthesia. He was instrumental in technology transfer to the next generation of surgeons representing varied specialties all over the world.

Rapid growth and popularity of this procedure across continents happened when Illouz,[1,3,9] a Plastic Surgeon from Paris, France, began favouring the “wet technique” in which a solution of hypotonic vasoconstrictor saline and hyaluronidase was infiltrated into the adipose tissue prior to aspiration. He termed this as a ‘dissecting hydrotomy’ which facilitated removal of fat and reduce trauma with less bleeding.

Lawrence Field[10] in 1977, a California dermatologist was the first American to visit France and learn the new field of liposuction. Norman Martin,[4] an otolaryngologist, visited Illouz in 1980 and began performing liposuction in Los Angeles in 1981. In 1982, physicians from different specialties were trained by Illouz and Fournier.[4]

In 1983 and 1984, several inter-specialty courses were held and Julius Newman,[4] an otolaryngologist was the first to use the term “LipoSuction”.[4] He went on to establish the American Society of Liposuction Surgery. The first articles on liposuction appeared in literature in July 1984.

Jeffery Klein, now at San Juan Capistrano, California, initially described the tumescent technique for lipo-aspirations in June of 1986 and the first article describing this technique was published in the January of 1987.[11] Ever since, Lipoaspirations and fluid managements have added a greater safety dimension. Ultrasonic liposuction was developed by Italian surgeon, Michael Zocchi[12] in 1996

Liposuction has evolved over the last 15 years with the introduction of the tumescent and super-wet techniques, ultrasonic assisted liposuction, power assisted liposuction and laser lipolysis. These advances have made possible the removal of larger volumes of fat with negligible blood loss and relatively trifle complications.

## INTRODUCTION

Liposuction is more of an art than a surgical procedure. It entails a practical application of scientific knowledge with precision and craftsmanship and is a skill attained with clinical experience. It brings as much contentment and joy to the person undergoing it, as to the surgeon practising the intimidating task of delivering that eventual result.

Liposuction for fat removal is similar to Phaco-emulsification of the ocular lens for cataracts. It permits elimination of localized fat deposits through miniature incisions that leave an inconspicuous scar. Principal indications are fat deposits in the gluteo-crural areas, hips and the abdomen. While the ideal body shape is trim and athletic, a well contoured shoulder and chest, a flat abdomen and a narrow hip and thigh area are sought-after and lipo-sculpturing is anticipated to bestow these expectations.

An increase in fat content can be either hypertrophic or hyperplastic. An increase in total fat cell numbers is hyperplastic obesity. It predominates as body fat levels exceed 40% and is more resistant to dieting and exercise regimens. In those cases where the actual number of fat cells remains stable, the cells increase or decrease in their volume with weight gain or loss.[13]

Localized fat accumulation patterns also vary by race and age. A decrease in the subcutaneous fatty layer and elevations in intra-abdominal fat contents are seen with increasing age. Women have a proportionately higher percentage of body fat than men and have a gynaecoid pattern of fat deposition characterized by increased deposits over the lateral thigh, buttock, hips, and truncal region, while men show an android pattern that centres on the truncal and abdominal regions.

Liposuction is effective in changing contour as it permanently removes fat cells that are unevenly distributed. The remaining adipocytes can still store fat, but to a smaller extent. For that reason, liposuction may not always (unless the amount removed is very large) prevent further weight gain but rather affects weight distribution.

**Figure 1:**
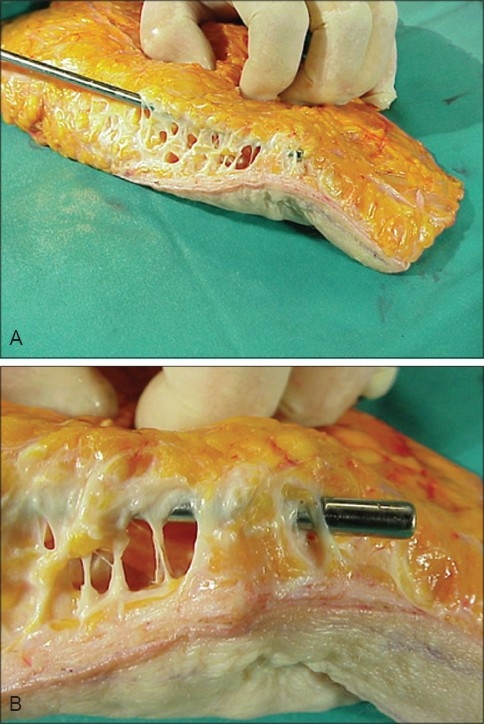
A Ultrasonic assisted Liposuction (UAL) with multiple tunnels & intact vertical septa

Fat in the trunk and extremities has a superficial and deep layer. The superficial layer is composed of small dense pockets of fat separated by vertical well-organized fibrous septa. The deeper fat layer is organized more loosely, with looser areolar fatty tissue interspersed with less regular fascial septae intervening between the pockets. Vertical septa originate from the fascia and extend upward toward the dermis [Figure 1]. These layers are important in avoiding potential complications during liposuction.

Suction lipectomy was initially advocated for the treatment of localized collections of fat and for the removal of less than 1500 ml of fat material. However, many patients wish to have multiple areas treated or have diffuse collections of fat. In such instances, the removal of over 1500 ml of material and circumferential lipectomy are necessary to present optimal aesthetic results. However, when over 1500 ml of material is removed, anaesthetic requirements, fluid replacement, and treatment of blood loss become important if the procedure is to be performed safely.

## TUMESCENT ANAESTHESIA

**Figure 2:**
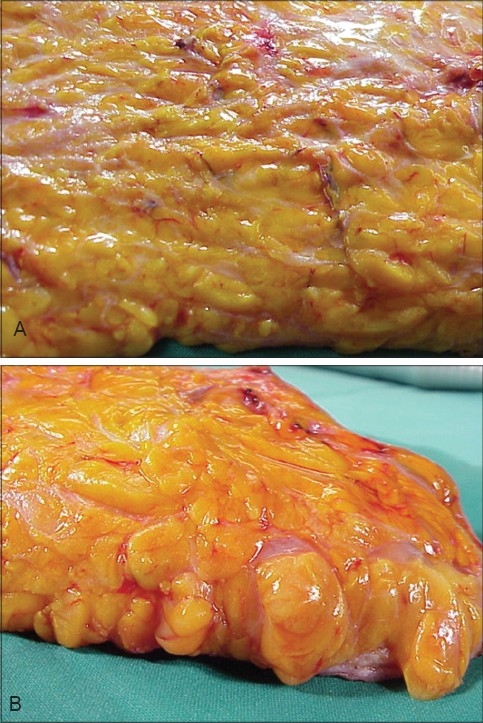
(A) Subcutaneous Fat, (B) Turgid Fat after Tumescent Infitration

Tumescence is the state of being ‘swollen and firm’ [Figure 2]. Tumescent liposuction uses large volumes of very dilute, hypotonic solutions of a vasoconstrictor agent that is gently injected into the subcutaneous fat and virtually eliminates surgical blood loss. It also permits the procedure to be done under regional anaesthesia with conscious sedation. Local anaesthesia may be supplemented for areas proximal to the level of the regional anaesthesia.

Limits of lignocaine dosage have been explored since the development of this technique. Lillis[14] unofficially reported no complications with tumescent lignocaine dosages of greater than 70 mg/kg.[4] Ostad *et al*,[15] proposed the maximum tumescent safe lignocaine dosage to be 55 mg/kg of body weight. Maximum safe dose of tumescent lignocaine was a major bone of contention in academic discussions. The demonstration that the peak lignocaine concentration in the blood occurs at approximately 12 hours of initiating of the tumescent infiltration as against the 2 hours as was originally conceived was an unprecedented finding. A safe dosage for tumescent lignocaine was shown to be 35 mg/kg to 50 mg/kg by Kleinin.[16] The rate of infusion of the tumescent anaesthesia was shown to be independent of plasma lignocaine levels.

For many years before the advent of the tumescent technique, general anaesthesia was an absolute requirement for liposuction along with the dry technique. Refinement and improvement of this technique over the years now allows liposuction to be done with exceptional finesse and gentleness and totally by regional and supplemental local anaesthesia.

The stinging pain originally associated with infiltration of local anaesthesia as a result of the acidic pH of commercially available Lignocaine has been eliminated by adding sodium bicarbonate to the anesthetic solution.

**Figure 3:**
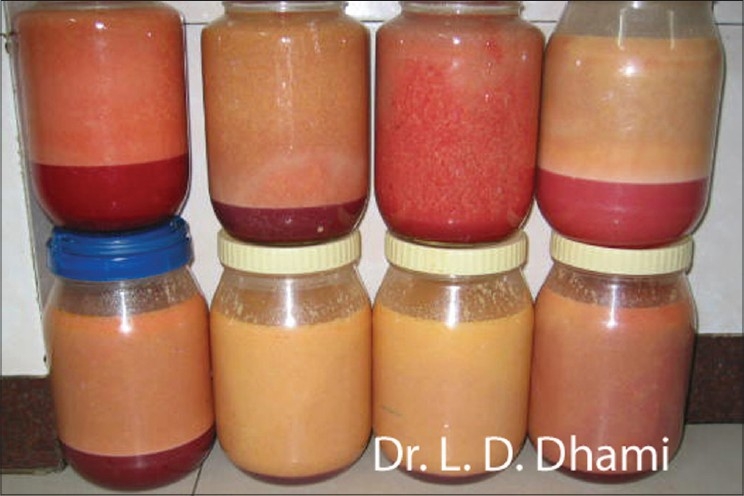
Total 24 litres of lipoaspiration with UAl & SAL combined including Fat + Wetting solution + Blood

The common definition of ‘large volume liposuction’ LVL refers to either total fat removed during the procedure or a total volume removed during the procedure (fat plus wetting solution). Because many of the complications associated with large volume liposuction are related to fluid shifts and fluid balance, classifying the procedure as large volume based on the total volume removed from the patient, including fat, wetting solution, and blood, is more acceptable [Figure 3].

Large volume liposuction clinically refers to the removal of more than 5 liters of total volume from the patient. Gilliland *et al*,[17,18] have appropriately segregated and better defined them as

1. Large Volume Liposuction (LVL) that is an aspirate of 5000 ml
2. Mega-volume liposuction as an 8000 ml aspirate
3. Giganto-volume liposuction as an aspirate of 12,000 ml or more because the safety and aesthetics issues differ at each level.

### Evolution of instrumentation

Initially, large cannulae were employed for liposuction, some even up to 1 cm in diameter. These instruments caused damage to neurovascular bundles and occasionally led to uneven contours and seromas or hematomas in patients. The subsequent use of local anaesthesia necessitated a gentle touch and hence a variety of smaller cannulae were developed.

The standard cannulae of the 1980's were huge, having diameters of 6 to 10 mm and cross sectional areas 9 to 25 times greater than today's 2 mm micro-cannulae. Illouz and Fournier[3,4,9] popularized liposuction using their newer generation of the blunt-tipped cannulae and the ‘wet technique’.

Cannulae used today are extremely small, typically less than 6 mm, some are very small with an inside diameter of less than 0.6 mm. Blunt-tipped cannulae are standard as they decrease injury to blood vessels and reduce bleeding. The use of multiple side ports allows for efficient evacuation of fat. Manual systems consisting of syringes and canula tips have also been developed as some surgeons prefer the use of quiet and disposable instruments, they are more popular in small local aspirations of isolated fat bulges. They also became popular as a back-up system. Over time, aspiration units developed by manufacturers in consultation with surgeons have gradually become more powerful as well as quieter and allow for an efficient, pleasant surgical environment.

Newer powered liposuction devices[19] for ‘Power Assisted Liposuction’(PAL) ^*^MicroAire employ reciprocating cannulae that facilitate fat removal and decreases the physical effort of the surgeon.

The initial ultrasonic liposuction started by the Europeans, emulsified fat with an ultrasonic canula which was then aspirated in the second step. This two stage procedure was time consuming. It was modified in the United States and now a modified suction canula emulsifies and aspirates the fat simultaneously.

While the proponents of the super-wet and tumescent techniques have their pros and cons open for discussion, most modern liposuction is a combination of these two techniques.

The task force of the American Society of Plastic and Reconstructive Surgeons (ASPS),[1,20] Plastic Surgery Education Foundation (PSEF), American Society for Aesthetic Plastic Surgery (ASAPS), Aesthetic Surgery Education and Research Foundation (ASERF) and the Lipoplasty Society of North America (LSNA) with Franklin Di Spaltro as the Chairperson, investigated Ultrasound Assisted Lipoplasty in 1995, evaluated the safety issues and provided inputs to the Food and Drug Administation (FDA) for its approval.

### Variations of liposuction

Ultrasound is used as an ablative tool in urology and neuro-surgery. Ultrasonic assisted liposuction (UAL) was developed and introduced in the early 1990s by Zocchi[12] in Italy. His interest in ultrasound was originally for harvesting collagen from aspirated fat. The chance observations that adipose tissues were effectively emulsified while connective tissue structures were preserved *in vitro* led to the concept of using ultrasound adjunctively in vivo.

The American Society of Plastic and Reconstructive Surgeons has promoted ultrasonic liposuction but surgeons of other specialties have abandoned this technique as they consider the internal ultrasound to increase the risk of cutaneous burns, seroma formation, and provide little additional benefit over standard liposuction.

Large volume tumescent liposuctions have gained acceptance in the West where the procedure is regularly practiced in large numbers. The author has initiated this trend in this country and advocates it. No major safety concerns have been noted in the series.[21]

### Other indications

**Figure 4:**
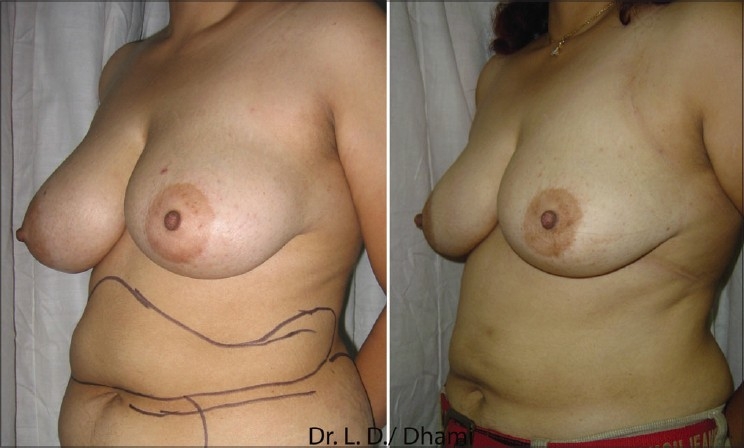
20 year old unmarried girl SAL Breast 650 ml each side. Before & after 3 months. She also had UAL Abdomen in same session

**Figure 5:**
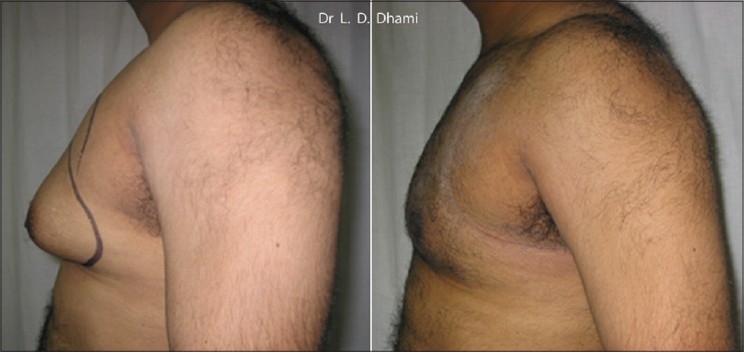
Pre and Post UAL Gynecomastia Combined Deep & Superficial LipoSuction

Non-cosmetic applications of liposuction were pioneered or developed by surgeons of other specialties. Liposuction could be used to remove lipomas, angiolipomas, and improve hyperhidrosis. Liposuction techniques can assist in hematoma evacuation. Klein[22] demonstrated liposuction techniques for breast reduction [Figure 4]. Field[23] pioneered liposuction to facilitate flap movement in cutaneous reconstruction, gynaecomastia, [Figure 5] and benign symmetrical lipomatosis (Madelung's disease), and Dercum's disease.

## MATERIALS AND METHODS

Megaliposuction in our series is defined as a volume aspirate of more than 10% of body weight in kgs. and includes the fat and wetting solution.

In the 8 years from September, 2000 to August, 2008. Suction assisted lipectomy (SAL) Ultrasonic assisted liposuction (UAL) or Power assisted liposuction (PAL) has been done for a total of 870 patients of whom megaliposuction was for 60% patients.

During this period, the youngest patient was 14 years of age and the oldest was 67 years old with a mean of 39 years. Individual patient weight ranged from 45 kg to 178 kg. with a mean of 78 kg Female patients dominate the series (86%) in a ratio of 7:1.

A combination of tumescent and the super wet liposuction were done simultaneously by two or three Plastic surgeons in multiple areas.

Prior to September 2002, the conventional tumescent liposuction technique was used. After September 2002, ultrasound-assisted liposuction was done for all patients by the same surgeons using the Ultrasonic Sonoca™ machine. After June 2008, we have used Power assisted Liposuction with MicroAire™ device in 29 Patients.

Blood transfusion was given only on one occasion when patient had been on aspirin and had also received Low Molecular weight Heparin intra-operative. Liposuction volumes between 5 and 25 liters (mean of 15.5 liters) were aspirated. Weight reduction 6 months post surgery at the patient's follow-up varied from 1 to 25 kg, with an average of 9.5 kg. 4% to 10% of preop body weight[21] Table 1.

**Table 1:** Average weight loss of patients at 6 months post operatively

	*Liposuction volume (in ml)*			
	
	*Conventional liposuction (SAL + UAL)*	*Megaliposuction - (SAL + UAL)*		
		
	< 5000	5,000 - 7,999	8,000 - 11,999	>12,000
Avg. Wt. Loss	4% of body weight	4%	7%	10%
at 6 months	(Range 2 to 6 kgs)	(Range 1 to 14 kgs)	(Range 2 to 25 kgs)	(Range 2 to 22 kgs)
	Avg - 3.2 kgs	Avg - 7.0 kgs	Avg - 9.5 kgs	Avg - 11.6 kgs

**Table 2:** Average tumescent fluid infiltration used

	*Liposuction volume (in ml)*			
	
	*Conventional liposuction (SAL + UAL)*	*Megaliposuction - (SAL + UAL)*		
		
	< 5000	5,000 - 7,999	8,000 - 11,999	>12,000
Average amount of fluid infiltrated	2 to 3.5 litres	2.5 to 6 litres	6 to 12.5 litres	1 to 2.5 litres

**Table 3:** Sequalae, complications and Management

*Sequelae / complication*	*Management*	
Immediate - noted up to 48 hours		
Pain	Conservative / Symptomatic	
Oozing		
Early - noted up to the first week		
Bruising, Ecchymosis, Swelling	Conservative / Symptomatic	
Late - noted up to six months		
	**Percentage**	
Seroma	Drained (1 to 3 sittings)	10.8
Necrosis	Debridement and secondary closure	2.4
Persistent induration with local rigidity	Local ultrasonic massage	1.7
Contour Irregularities	Local ultrasonic therapy + revision surgery	5.7

SAL + block abdominoplasty / mini abdominoplasty were carried out in 24% of the patients. The duration of the surgical procedure ranged from 2 to 3.5 hours with a minimum of two surgeon team.

**Figure 6:**
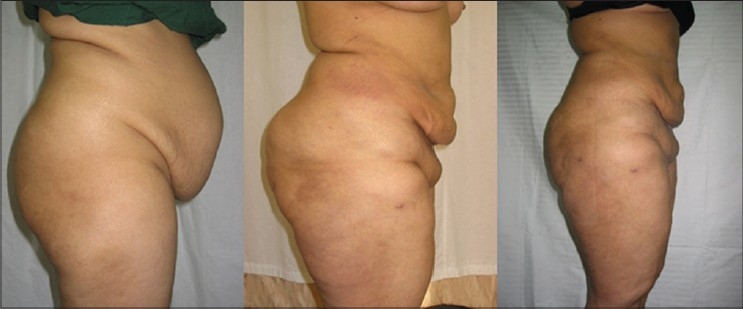
28 years old unmarried girl (A) pre op (B) 6 months after first stage large volume liposuction (12000 ml) of Abdomen & Antero-medial thigh (C). 6 months after secong stage LVL (9000 ml) of Buttocks, Back & Lat Thigh

**Figure 7:**
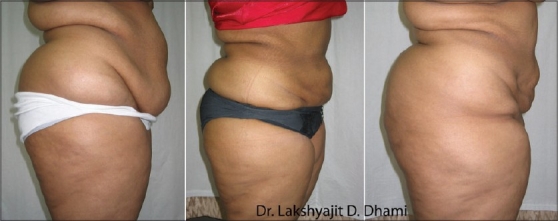
27 years old unmarried girl with MALL (massive all layer liposuction). Total 32,000 ml in 2 session (A) Pre operative (B) 5 months after UAL+SAL of Abdomen, antero-medial thigh & Medial Arms −Anterior Torso (14,000 ml) (C) 9 months after 2nd session of UAL+SAL from Back, Buttocks & PosteroLateral thigh −Posterior Torso (18,000 ml)

10% patients though content with the results of first liposuction, sought a second SAL after a time interval of 7 days to 6 months from the primary procedure for additional areas [Figures 6–7].

### Post operative care

Early ambulation within 24 hours was encouraged for mobilization of third space fluid shifts to expedite recovery and to prevent deep vein thrombosis. Prolonged sitting is to be avoided for 3 to 4 weeks following abdominal liposuction and pressure garments are to be worn for 3 to 6 months. It is important to support the heavy skin and subcutaneous fat of the obese patient longer than advocated to prevent it from gravitating down and forming folds, because skin retraction takes longer following megaliposuction. Pressure (finger tip) massage or an external ultrasound massage is advised for persistent edema, pain or firm and lumpy areas.

## SURGICAL TECHNIQUE

### Preoperative markings

Precise and accurate pre operative marking is essential for a good result. With the patient standing, areas to be treated are outlined with a fiber tip permanent marker pen. Areas to be avoided or areas for fat grafting are also separately identified. Port sites per area are defined to allow cross-tunnelling aspiration to minimize surface abnormalities.

### Preparation and positioning

The patient is prepared circumferentially in the torso and the lower extremity as these can be treated without repeated pepping and repositioning. The patient's skin is painted with 5/10 percent Povidone Iodine solution while he/she stands next to a sterile draped operating table. Upon completion of the skin preparation, the patient lies on the table and is sedated or is given regional or general anaesthesia as required.

### Tumescent infiltration

All areas to be treated are injected with large volumes of a diluted epinephrine solution till turgour of the tissues is appreciable equally on both sides. Effective vasoconstriction is achieved in about ten to fifteen minutes, but the effect is more pronounced after about twenty minutes.

Tumescent fluid (maximum of 12,500cc in the series)

**Table:** 

Ringer lactate (RL)	1000 cc
Inj. adrenaline	2 amp
Inj. hyalase	1 amp
Inj. triamcinolone	10 mg

**Figure 8:**
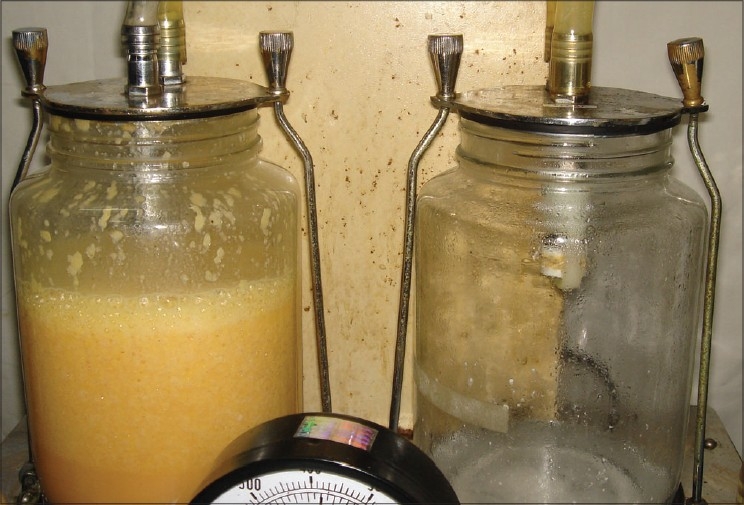
Yellow Fat aspiration with insignificant Blood after tumescent infiltration

Physiological saline was used in the place of RL previously. The author has noted an appreciable reduction in the tissue swelling post operatively after shifting to RL solution in place of conventional normal saline. The hypotonic saline solution results and Ringer Lactate results were identical and hence the current method of Infiltration fluid does not have any hypotonic saline fluid. The intense local vasoconstriction reduces blood loss to insignificant amounts for most procedures [Figure 8].

### Aspiration

Access incisions of size 1.5 cm are made at the periphery of operative field in concealed areas and are used separately for all areas as removing all the fat from a single incision may lead to a depression around the access site.

Aspiration begins 20 minutes after infiltration. Deeper areas and areas with more voluminous fat deposits are aspirated using cannulae of 5 or 6 mm diameter. Smaller fat deposits and the more superficial areas are aspirated with cannulae 3 to 4 mm in diameter.

The cannulae move parallel to the fat plane with the openings directed away from skin surface in a to and fro motion along the same path. The site is changed when the aspirate tends to become blood stained. Feathering of the peripheral areas is done once the basic earmarked areas have been symmetrically contoured bilaterally. The closure of these access incision sites is accomplished with interrupted loose sutures to permit easy drainage of fluid, reduce oedema and seroma.

The end point of aspiration is determined by the contents and volume of aspirate as also the appearance and feel of the treated area and bilateral symmetry. Aspirate volumes from bilaterally symmetrical areas should be approximately the same, although the volume of the preoperative injection will influence the volume of the aspirate.

Caution is to be exercised in the learning curve when shifting to the UAL, or by the beginner aesthetic plastic surgeon to minimize concurrent damage to the vital structures and damage to the overlying skin.

### Technique and instrumentation

**Figure 9:**
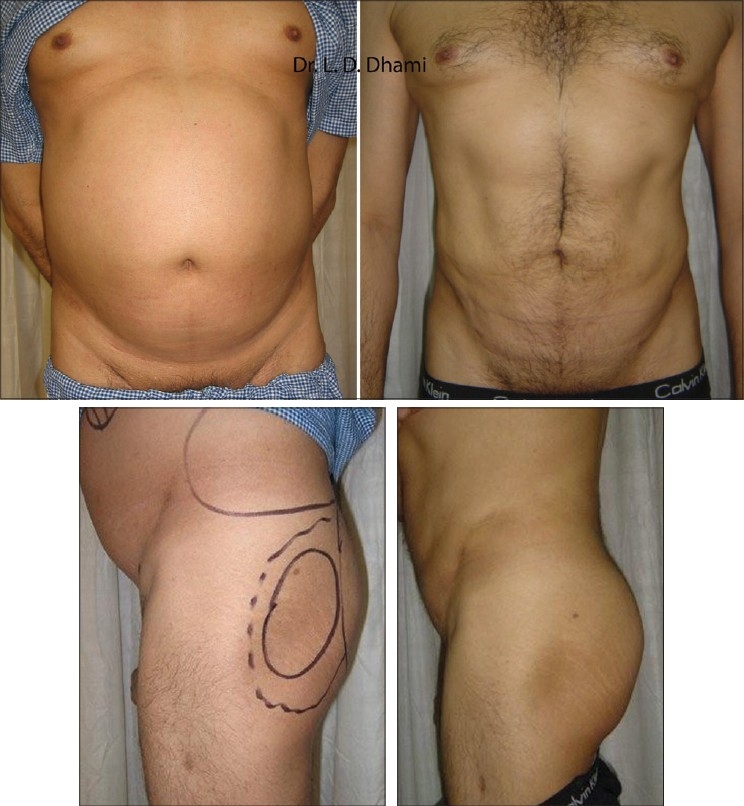
48 year old man before & 1 year after SAL of Abdomen & Flanks. Fat was used as Transplant for Buttocks augmentation

Although it is not essential to suction the sub-dermal layer of fat in large-volume lipoaspiration, the author concurs with the Massive All Layer Liposuction Mall[24] concept as it helps reduce the thickness and consistency of the superficial fat and enhances skin retraction. This however is better indicated in cases where there is only a limited correction for body contouring rather than volume reduction [Figure 9].

**Figure 10:**
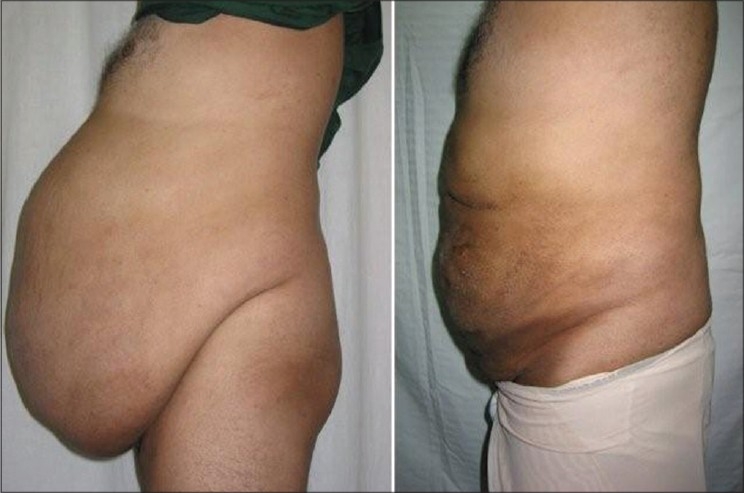
Before & 6 weeks After UAL of Abdomen. 47 year old man with MALL of 20,000 ml

**Figure 11:**
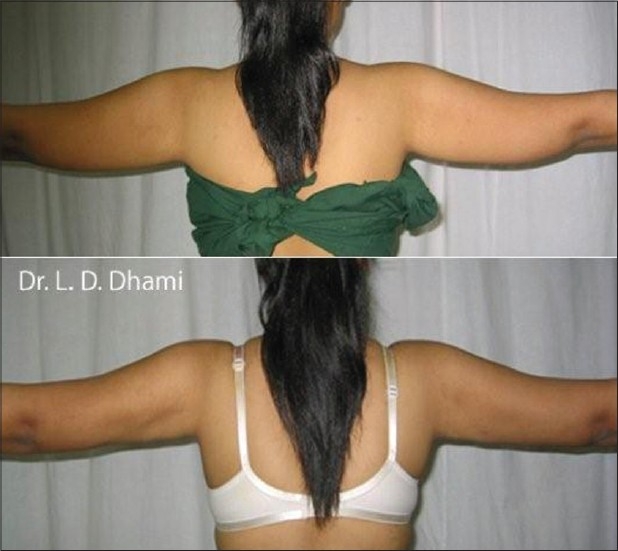
Before & 4 months after SAL of medial arms in 29 year old girl. Good Skin Contaction even with SAL alone

Large adiposity of the abdomen, arms, or inner thighs tends to have excess volume of fat whose weight overstretches the panniculus and results in a ptosis of the skin overlying the area. In these cases the need is to reduce the large fat volume to permit effective skin retraction and MALL effectively addresses the issue better as the amount of skin shrinkage after this procedure is remarkable and the clinical results are appreciable [Figures 10–11].

## DISCUSSION

For the obese individual, a safe and limited surgical intervention that achieves even a minimally acceptable aesthetic contour of their profile in proportion to the body structure greatly enhances their self esteem. This is the prime indication and forms the essence of the much touted large volume liposuction. In most instances, the technique may be combined with a block dermolipectomy.

Many Plastic surgeons are still apprehensive about the physiology of large-volume liposuction and patients being exposed to prolonged procedures, anaesthesia, fluid shifts, and infusion of high doses of epinephrine and Lignocaine. The super-wet and tumescent techniques used under regional anaesthesia permits local anaesthesia of the skin and subcutaneous tissues by direct infiltration. Large volumes of a lactated Ringer's solution with epinephrine and the limited use of dilute anaesthetic solutions produce tumescence and firmness of targeted areas. Dilution of lignocaine and epinephrine diminishes and delays their peak plasma concentrations reducing potential toxicity.

General Anaesthesia was used previously when anaesthesiologists were unaware that the FDA limits on Lignocaine were designed exclusively for epidural anaesthesia (7 mg/kg) and that the limits for tumescent local anaesthesia are much higher (35 mg/kg).[16]

### Spinal anaesthesia

Spinal anaesthesia is preferred as bulk of fat to be sucked out is situated in lower half of the torso and the duration of surgery is about three hours. It reduces the drugs administered to the patient as general anaesthesia is avoided. A 27 gauge needle avoids the often troublesome post spinal anaesthesia headaches. Fentanyl is additionally added to the high spinal because it has a bupivacaine sparing effect on spinal anaesthesia. This covers the subcostal areas as well and further reduces the need of lignocaine in the infiltrating solution.

### Intravenous fluids in large volume liposuctions

The fluids administered are by clinical judgment, keeping an eye on the clinical monitors of the pulse rate, blood pressure as well as the colour and amount of urine collected with indwelling Foley's catheter.

Administered fluid amount is normally calculated as a sum of the normal requirement under anaesthesia of 8 ml/kg/hr, 1000 ml crystalloids for an 8 hour starvation and compensate blood loss if any, which is more than anticipated or expected.

Considering these factors, in megaliposuctions where about 10 litres or more of fat and wetting solution is aspirated in an otherwise fit patient and with the surgery lasting for about 3 hours the following factors need to be considered.

1. Total intravenous (IV) fluid usually given to a patient under General Anaesthesia is:
  2000 ml crystalloid + 1000 ml artificial colloids/plasma expander. Depending on the clinical parameters, the rate of the fluid is adjusted accordingly.
2. Total IV fluid usually given to a patient under Spinal Anaesthesia:
  1000 to 1500 ml crystalloids administered pre operatively as a priming solution and another 2000 ml of crystalloids given at the time of SA. Here again, depending on the clinical parameters, the rate of the fluid is adjusted accordingly. Overall, the patient, under Spinal Anaesthesia, will need about 1500 ml of crystalloids and 500 ml of colloid more than required under general anaesthesia.

### Local anesthetic in the tumescent solution

The aim is not to exceed the toxic dose of the drug in mg/ml/kg body weight. A single drug alone would exceed the toxic level because it would be needed in a large amount. This is addressed by:

- Using a larger quantity in a lower concentration i.e. instead of using 10 ml of a 2% solution, it is advisable to use 40 ml of a 0.5% solution.
- Adding two drugs with different toxicity, (i.e. lignocaine that causes Central Nervous System depression or stimulation and bupivicaine that is cardiotoxic.)

The tumescent infiltration solution is additionally added with the local anesthetic only in the terminal portion which is to be used for infiltration between the supra-umbilical and the subcostal areas when the patient is under spinal anaesthesia/epidural anaesthesia. This solution contains:

**Table:** 

1.	Ringer lactate	1000 cc
2.	Inj. adrenaline	2 amp
3.	Inj. hyalase	1 amp
4.	Inj. triamcinolone	10 mg
5.	Inj. xylocaine 2%	40 ml
6.	Inj. bupivacaine 0.5%	20 ml
7.	Inj. soda bicarbonate	40 ml

A 40 ml solution of 2% lidocaine is 800 mg of the drug. As the toxic dose of lidocaine is 35 mg/kg when used in the tumescent fluid, it is safe to infiltrate 3.5 to 4.5 litres, when used for a patient of 80-100kg body weight.

### Adrenaline

Each 1000 ml of the tumescent fluid has 2 amp of (1:1000) adrenaline. Thus, even when 25 ampoules of the adrenaline were used in the maximum infiltration of 12,500 cc,, no side effects or complications attributed to the large dose of adrenaline used have been noticed in the entire series over the past 8 years. Because adrenaline causes vasoconstriction which prevents sudden absorption of more adrenaline till its effect has waned. Systemic toxic effects of this drug are not seen.

Tumescent liposuction has proven to be extremely safe even with the use of unprecedented large doses of the tumescent solution with dilute epinephrine, this produces intense widespread capillary constriction in the targeted fat, which in turn greatly delays the rate of absorption of the drug. This diluted epinephrine is absorbed into the bloodstream over 24 to 36 hours. This reduces peak concentration of the drug in the blood, which in turn reduces its potential receptor stimulant actions.

The profound vasoconstriction is so absolute that liposuction can be done with virtually no blood loss. In contrast, the older forms of liposuction used before the invention of the tumescent technique was associated with much surgical blood loss that autologous blood transfusions were often a routine.

**Figure 12:**
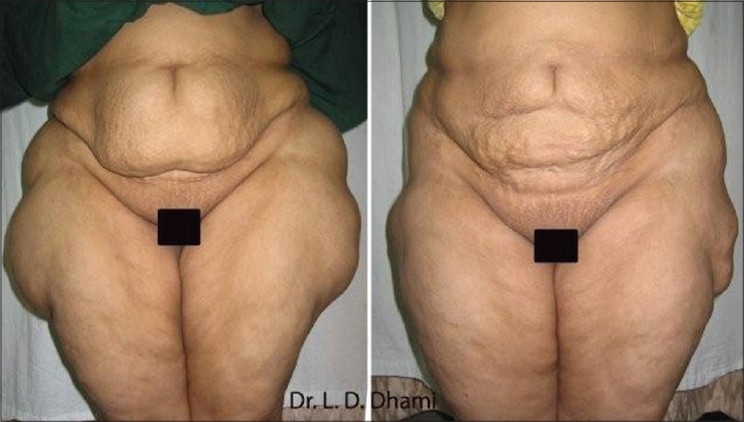
55 years old Female with UAL of 8000 ml from Abdomen & Lateral Thigh (A) Before and (B) 1 year after

Clinically, the skin should have sufficient inherent elasticity to recoil and contract after removal of fat. Stretch marks are a strong indication of poor elasticity, as is delayed rebound after manual stretching. Significant skin overhang indicates a need for adjunctive surgical procedures [Figure 12].

Access incision sites are small and it is advisable not to close them with sutures to permit drainage of the excess wetting solution and serous exudate. Larger canullae require larger incisions, but these must be sutured loosely. A delayed drainage of the blood tinged tumescent solution produces prolonged swelling, bruising and pain after liposuction.

Larger cannulae remove fat rapidly and there is a risk of removing too much fat and produce skin depressions and irregularities. An attempt to make a small change in the direction with a large canula results in a tendency to re-enter a pre-existing tunnel within the fat. This lack of precise control results in skin irregularities associated with the use of large cannulae. They are advocated only in those cases of LVL with access from sites from where the panniculus is to be sacrificed.

Microcannulae with an external diameter of 4mm can remove fat very efficiently and are effective in achieving a smoother liposuction as they allow for a more gradual and controlled removal of fat.

Pre-tunnelling (Mladick)[25] increases instrument control as, without suction, it creates desired superficial as well as deep planes of fat removal. It prevents an inadvertent removal in the sub-dermal fat layer that can result in contour irregularities. Similarly, cross tunnelling with at least two port sites at right angles are used to treat an area of adiposity. The use of multiple port sites provides for better contouring and feathering of edges.

Fat layers are treated from deep to superficial in sequence and in parallel tracks. As the procedure is progresses more superficially, canula size can be decreased along with reduction in suction intensity to help decrease risk of irregularity to the surface layers. Most traditional liposuction treatment involves removal of the deeper layers of fat. Superficial liposuction is done in individuals with flaccid skin or Localised Fat Deposits (LFDs) as an aid to better skin retraction. It is accomplished with narrow cannulae that make multiple closely spaced passes in the sub-dermal fat to effect an undermining of the affected tissue.

Symmetry (if bilateral), skin pinch of less than 1 inch, shape and an overall smooth contour determines the clinical end points of the procedure. Further removal of the remaining fat gives the advancing canula a grittier feel as it passes in the tunnels against the remaining fibrous septae.

Port sites if contused, are re-excised to improve scar, and closure is done with a loose deep dermal absorbable suture. Absorbent materials like gamgee or presterilised sanitary pads are applied to prevent spoilage of the compressive binders and dressings.

### Ultrasonic assisted liposuction (UAL)

Ultrasonic techniques are available as an internal, canula based, as well as an external one, with paddle application. The high ultrasonic energy produced by passing electrical energy to a piezoelectric crystal creates micro cavities in a liquid or semi liquid medium during the expansion cycle of the sound wave. This property of microcavitation is used in UAL.

Zocchi[12] states that the susceptibility of a liquid or biologic tissue to microcavity formation depends upon the molecular cohesion of the material and that the negative pressure required is related to the density of the tissue for its aspiration. Low-density tissues such as fat cells have low molecular cohesion, and this favours micro cavity formation and aspiration.

Connective tissue and muscle are essentially unaffected by this process as they are more dense but an accumulation of secondary thermal energy and micromechanical trauma on sustained application of ultrasound after complete emulsification by microcavitation may result in damage. Thus direct micromechanical trauma and secondary thermal effects of persistent ultrasound energy is the mechanism of action for external UAL.

**Figure 13:**
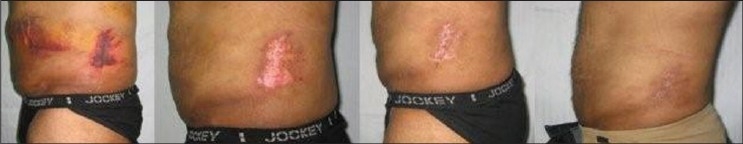
Complication. Ecchymosis, Superficial Skin necrosis after UAL & it's spontaneous resolution with conservative treatment over 6 weeks

There is an enhanced fat removal with minimal blood loss, improved skin retraction and safer large-volume procedures with the UAL. Reports of cutaneous burns, [Figure 13] hypo and hyperaesthesia and seroma formation brought considerable debate concerning the long-term effects and clinical use of UAL. Subsequent evidence of large trials with long term follow-ups have led it to be well-established and accepted technique.[26,27] It is especially indicated in areas of dense, fibrotic fat.

**Figure 14:**
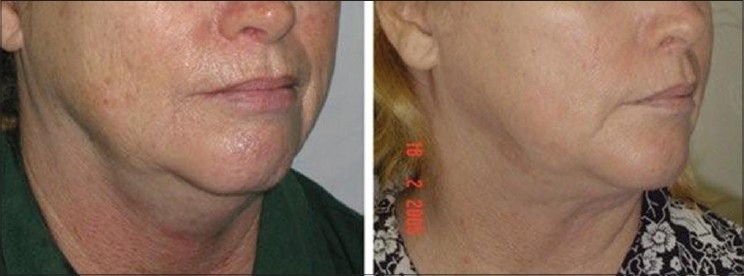
58 year old female. SAL Double chin, Before & After 3 months shows good skin contraction

**Figure 15:**
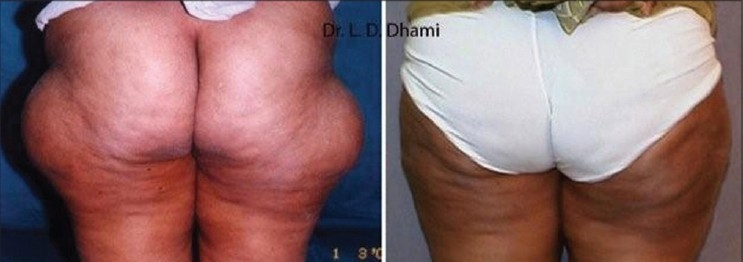
43 year old female with Before & After SAL of 1500 ml from Bilateral Trochanteric region

Inner knee, medial thigh or submandibular region [Figure 14] with a less dense fat are better managed with a standard wet technique rather than the UAL. Improved results with less fatigue in treating fibrous areas such as gynecomastia, posterior trunk, upper abdomen, posterior hip rolls and trochanteric regions [Figure 15] support the use of UAL as an adjunct to SAL rather than as an alternative.

UAL is presumed to enhance skin retraction by a controlled thermal stimulation of the dermal collagen. The results with the use of UAL in large-volume liposuction in patients with lax skin have been good and in this series have even shown to decrease concurrent lipectomies.

### Power assisted liposuction (PAL)

PAL technique is based on power assisted reciprocating movements of the cannulae. The advantage being, the surgeon is not fatigued with rapid to and fro movements of cannulae and can concentrate on refinement. It also allows the use of finer cannulae and the results are better. Some tough tissues like in Gynecomastia are relatively easily suctioned and Liposuction in female breast, (where UAL may have unproven potential for thermal damage to mammary gland) can also be managed.

## COMPLICATIONS AND CAUTION

An unsatisfied patient as a result of unrealistic expectations prior to surgery is by far the most common problem. Careful and accurate communication between patient and surgeon helps the patient make a well-informed decision and obviates many a ‘fact justifying’ consultation in the post operative period.

**Figure 16:**
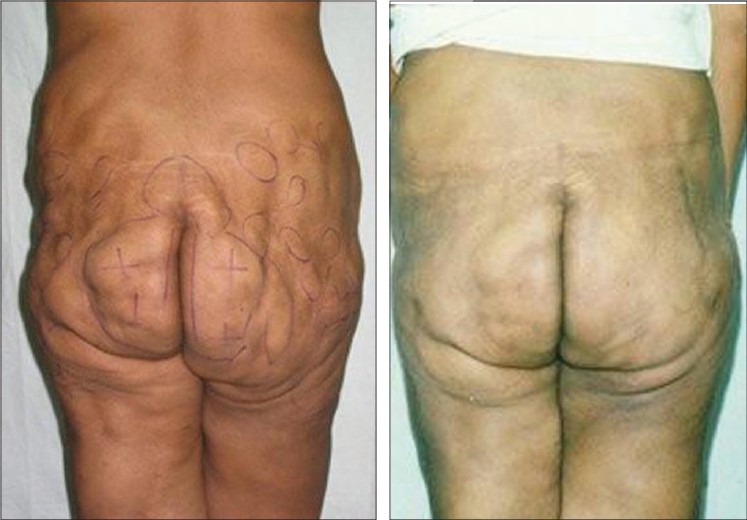
Complication: (A) Overzealous SAL, Scooping leading to ugly deformity. (B) Fat-filling done to correct

The access incision when placed at the centre of the operative field may leave a residual bulge or a crater at that location. A side to side canula movement may result in scarring, surface irregularities or skin necrosis while an overzealous correction ends up with a scooped effect and would probably need an additional correction with fat graft at a later date if the patient so desires [Figure 16].

Care must be exercised in the gluteal crease, lateral gluteal depression, distal posterior thigh, middle medial thigh, and the infero-lateral ilio-tibial band as these areas have an increased susceptibility to superficial contour deformities due to minimal amounts of deep fat and adherence of the more superficial layer to the underlying fascia or the muscle.

**Figure 17:**
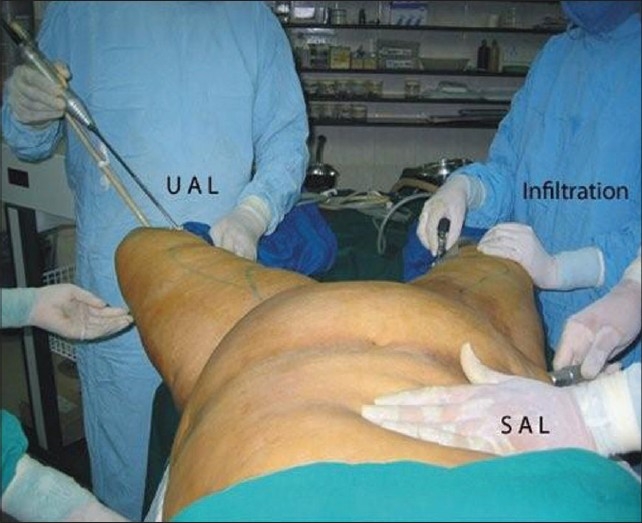
Team work - Simultaneous Infiltration, UAL & SAL by 3 surgeons leading to reduction in duration of surgery for large volume liposuction

Team work, a judicious and an appropriate selection of a surgically and medically fit patient are essential factors resulting in an overall reduction in the duration of the surgery to within three hours [Figure 17]. When a large volume liposuction is planned for an obese patient, it is advisable to stage this procedure in 2 to 3 session. It is preferable to perform liposuction on either front of the torso in supine position or the back in prone position. This avoids the need to change the position, or turning the patient over, half way through the operation, thereby taking additional time. This also reduces the patients' exposure to the rigorous physiological demands of this procedure.

Per operative, suction with a slow and regular canula motion in addition to an adequate pre-tunnelling with fine to small cannulae achieves desired results with a minimal morbidity to the patient and is less taxing to the surgical team.

**Figure 18:**
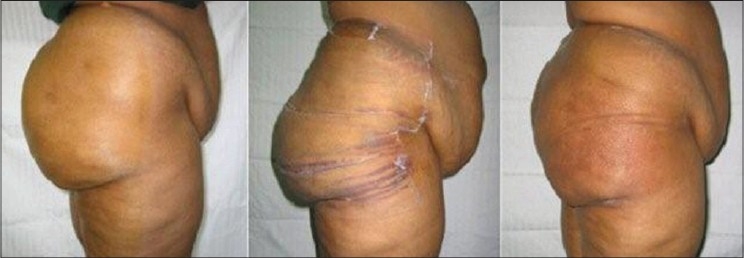
40 year-old female. Pre, 5th Post op & 3 months post UAL (5500 ml) Buttocks. Good Skin retraction following Ultrasonic Liposuction

Though suction assisted lipoplasty SAL achieves almost similar results, author's experience with the UAL has shown that a better skin shrinkage and retraction is achieved with the UAL by virtue of its physical collagen stimulation action [Figure 18]. This energy not only helps to break the turgid adipocytes more easily, but it also helps, in less strenuous to and fro movement, in areas of fibrotic fat. It is our observation that male obesity is more often associated with tougher adipocytes, and hence liposuction with UAL becomes easier. UAL also requiring less physical exertion for the surgeon, more attention can be given to the sculpturing rather than the mechanical process itself.

Patients are encouraged to ambulate on the same day to prevent deep vein thrombosis. While this obviates the need to administer the heparin prophylaxis, it also achieves an improved intra-space fluid shift which facilitates an early recovery of the patient and discharge on the same day.

In the post operative period following abdominal liposuction, the patients are advised to avoid prolonged sitting for 3 to 4 weeks to prevent development of skin folds and creases. Pressure garments are to be religiously worn for 3 to 6 months. While, a regular finger tip pressure massage in the indurated areas is fine with small areas of induration, an ultrasound massage works well for lumpy areas and sites with persistent pain or edema.

### Complications

**Figure 19:**
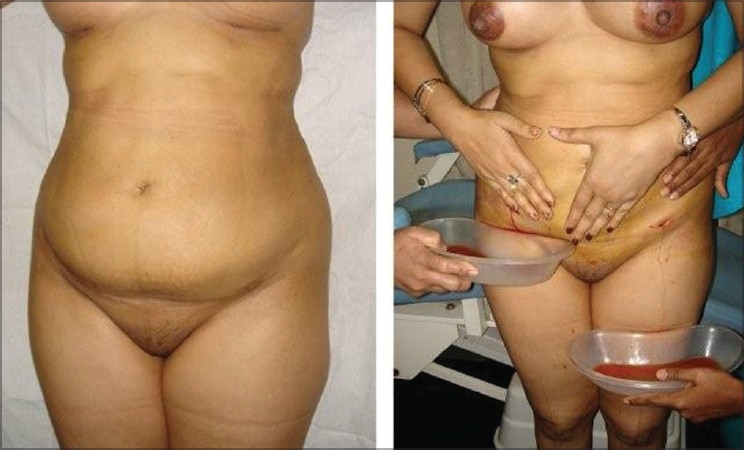
Compication: Large Seroma after UAL. 7th postop day. Drainage from incision site, left open for further drainage

Complications noted after some of the liposuction procedures are conditions that include superficial irregularities of the skin, seroma, [Figure 19] haematoma, focal superficial skin necrosis, allergic reactions to drugs or sticking plaster, visible or disfiguring scars, discoloration of the skin, temporary bruising, numbness or nerve injury and temporary adverse drug reactions. These complications do make the patient function at a sub optimal level, but have not been noted to disturb the normal routine in the post operative phase. Post-liposuction standing, leading to postural hypotension and syncope during the first 8 to 12 hours is not rare and patients need to be cautioned in this regard.

The sequalae as and when they occur, most commonly resolve satisfactorily with the suitable management.

## CONCLUSION

Liposuction can be performed safely on properly selected patients who have realistic expectations and understand the limitations of the procedure. They tend to be very satisfied with the results.

Motivation, goals, and expectations of the patient must agree with what is clinically possible. Patients should be psychologically stable with good diet and exercise habits or evidence of motivation toward them. The procedure is ideally performed at accredited centres with appropriate equipment and well trained staff.

The surgeons who perform liposuction must understand the physiology and differences between smaller volume and large volume liposuction. The anaesthesiologist is an integral member of the team and must have a complete understanding of the procedure and be well trained to handle preoperative, peri-operative, or post-operative problems of fluid shits and drug toxicity. The patient's core body temperature must be maintained using heating blanket systems on the table, minimizing body exposure and using warmed wetting solution.

It is to be appreciated and emphasised that the dreaded complications of pulmonary embolism, deep venous thrombosis, penetration injuries, bleeding, pulmonary oedema, hypovolemic shock, fat emboli, drug toxicity and mortality are absent in every large series of large volume liposuction. In all these cases, credit goes to a strict adherence to the 5 pillars of safety (safe surgeon, safe anaesthesiologist, safe facility, safe co-workers and a properly selected patient).

The super-wet technique of fluid infiltration is used to maintain an almost bloodless aspirate. Compressive pressure garments are always worn in the immediate postoperative period to keep skin in close contact with underlying muscle and prevent any dead space. This helps to minimize postoperative bleeding, serous oozing, swelling and a third space shift of fluid.

The long-term results of liposuction depend on the preoperative condition of the patient's skin, the patient's overall health and expectations and the ability of the patient to maintain a healthy weight and lifestyle postoperatively. In difficult cases and for large volume liposuction, it is prudent to be wise and a staged or a combined procedure is safer for both the patient as well as the surgeon.

Large volume liposuction is advocated as therapeutic body contouring in the excessively obese, well motivated and physically fit patients. When meticulously executed as a standard operative procedure, it carries negligible risks and maximises the eventual realistic aesthetic and functional gains.

## References

[1] Illouz YG (1983). Body contouring by lipolysis: A 5-year experience with over 3000 cases. Plast Reconstr Surg.

[2] Coleman WP (1999). The history of liposuction and fat transplantation in America. Dermatol Clin.

[3] Illouz YG (1996). History and current concepts of lipoplasty. Clin Plast Surg.

[4] Flynn TC, Coleman WP, Field LM, Klein JA, Hanke CW (2000). History of liposuction. Dermatol Surg.

[5] Grazer FM, deJong RH (2000). Fatal outcome from liposuction: Census survey of cosmetic surgeons. Plast Reconstr Surg.

[6] Pitanguy I (1964). Trochanteric lipodystrophy. Plast Reconstr Surg.

[7] Kesselring UK, Meyer R (1978). A suction curette for removal of excessive local deposits of subcutaneous fat. Plast Reconstr Surg.

[8] Fournier PF, Otteni FM (1983). Lipodissection in body sculpturing: The dry procedure. Plast Reconstr Surg.

[9] Illouz YG (1984). Illouz's technique of body contouring by lipolysis. Clin Plast Surg.

[10] Field LM (1987). The dermatologist and liposuction: A history. J Dermatol Surg Oncol.

[11] Klein JA (1987). The tumescent technique for liposuction surgery. Am J Cosm Surg.

[12] Zocchi M (1998). Ultrasonic-assisted lipoplasty. Adv Plast Reconstr Surg.

[13] Wagner BM (1985). Adipose tissue and obesity. Hum Pathol.

[14] Lillis PJ (1988). Liposuction surgery under LA: Limited blood loss and minimal lidocaine absorption. J Dermatol Surg Oncol.

[15] Ostad A, Kageyama N, Moy RL (1996). Tumescent anaesthesia with a lidocaine dose of 55 mg/kg is safe for liposuction. Dermatol Surg.

[16] Klein JA (1990). The tumescent technique for regional anaesthesia permits lidocaine doses of 35 mg/kg for liposuction. J Dermatol Surg Oncol.

[17] Gilliland MD, Coates N (1997). Tumescent liposuction complicated by pulmonary edema. Plast Reconstr Surg.

[18] Gilliland MD, Commons GW, Halperin B (1999). Safety issue in ultrasound assisted large volume Lipoplasty. Clin Plast Surg.

[19] Katz BE, Bruck MC, Felsenfeld L, Frew KE (2003). Power liposuction: A report on complications. Dermatol Surg.

[20] Albin R, de Campo T (1999). Large volume liposuction in 181 patients. Aesthet Plast Surg.

[21] Dhami LD, Meenakshi A (2006). Safe total corporal contouring with large volume liposuction for obese patient. Aesthet Plast Surg.

[22] Klein JA (1995). Tumescent technique chronicles: Local anaesthesia, liposuction and beyond. Dermatol Surg Oncol.

[23] Field CM, Skouge J, Anhalt TS, Recht B, Okimoto J (1988). Blunt liposuction cannula dissection with and without suction assisted lipectomy in reconstructive surgery. J Dermatol Surg Oncol.

[24] Gasperoni C, Salgarello M (1994). Mall Liposuction: The natural evolution of subdermal superficial liposuction. Aesthet Plast Surg.

[25] Mladick RA (1989). The big six: Six important tips for a better result in Lipoplasty. Clin Plast Surg.

[26] Graf R, Auersvald A, Damasio RC, Rippel R, de Araujo LR, Bigarelli LH (2003). Ultrasonic assisted liposuction: An analysis of 348 cases. Aesthet Plast Surg.

[27] Omranifard M (2003). Ultrasonic liposuction versus surgical lipectomy. Aesthet Plat Surg.

